# School Outcomes Among Children Following Death of a Parent

**DOI:** 10.1001/jamanetworkopen.2022.3842

**Published:** 2022-04-08

**Authors:** Can Liu, Alessandra Grotta, Ayako Hiyoshi, Lisa Berg, Mikael Rostila

**Affiliations:** 1 Department of Public Health Sciences, Stockholm University, Stockholm, Sweden; 2 Centre for Health Equity Studies, Stockholm University/Karolinska Institutet, Stockholm, Sweden; 3 Clinical Epidemiology Division, Department of Medicine, Solna, Karolinska Institutet, Stockholm, Sweden; 4 Clinical Epidemiology and Biostatistics, School of Medical Sciences, Örebro University, Örebro, Sweden; 5 Department of Epidemiology and Public Health, University College London, London, England

## Abstract

**Importance:**

To better support children with the experience of parental death, it is crucial to understand whether parental death increases the risk of adverse school outcomes.

**Objectives:**

To examine whether parental death is associated with poorer school outcomes independent of factors unique to the family, and whether children of certain ages are particularly vulnerable to parental death.

**Design, Setting, and Participants:**

This population-based sibling cohort study used Swedish national register-based longitudinal data with linkage between family members. Register data were collected from January 1, 1990, to December 31, 2016. Data analyses were performed on July 14, 2021. The participants were all children born between 1991 and 2000 who lived in Sweden before turning age 17 years (N = 908 064).

**Exposure:**

Parental death before finishing compulsory school.

**Main Outcomes and Measures:**

Mean school grades (year-specific *z* scores) and ineligibility for upper secondary education on finishing compulsory school at age 15 to 16 years. Population-based cohort analyses were conducted to examine the association between parental death and school outcomes using conventional linear and Poisson regression models, after adjustment for demographic and parental socioeconomic and health indicators measured before childbirth. Second, using fixed-effect linear and Poisson regression models, children who experienced parental death before finishing compulsory school were compared with their siblings who experienced the death after. Third, the study explored the age-specific associations between parental death and school outcomes.

**Results:**

In the conventional population-based analyses, bereaved children (N = 22 634; 11 553 boys [51.0%]; 11 081 girls [49.0%]; mean [SD] age, 21.0 [2.8] years) had lower mean school grade *z* scores (adjusted β coefficient, −0.19; 95% CI, −0.21 to −0.18; *P* < .001) and a higher risk of ineligibility for upper secondary education than the nonbereaved children (adjusted risk ratio, 1.36; 95% CI, 1.32-1.41; *P* < .001). Within-sibling comparisons using fixed-effects models showed that experiencing parental death before finishing compulsory school was associated with lower mean school grade *z* scores (−0.06; 95% CI, −0.10 to −0.01; *P* = .02) but not with ineligibility for upper secondary education (adjusted risk ratio, 1.07; 95% CI, 0.93-1.23; *P* = .34). Independent of birth order, losing a parent at a younger age was associated with lower grades within a family.

**Conclusions and Relevance:**

In this cohort study, childhood parental death was associated with lower school grades after adjustment for familial confounders shared between siblings. Children who lost a parent may benefit from additional educational support that could reduce the risk of adverse socioeconomic trajectories later in life.

## Key Points

**Question:**

Is parental death associated with increased risk of adverse school outcomes after accounting for familial confounders shared between siblings?

**Findings:**

In this cohort analysis of the nearly 1 million children in the Swedish national birth cohort of 1991 to 2000, children exposed to parental death before finishing compulsory school had statistically significantly lower school grades, but no difference in ineligibility for upper secondary education, compared with their siblings exposed to parental death after finishing compulsory school.

**Meaning:**

Bereaved school-aged children may need additional educational support.

## Teaser

This cohort study investigates the association between parental death and school outcomes using the Swedish national birth cohort of 1991 to 2000.

## Introduction

Parental death in childhood is associated with long-term ill health.^1,2^ Among many health, behavioral, and social consequences after parental death,^3^ high-risk behavior in adolescence and low socioeconomic status in adulthood are important underlying mechanisms.^4^ Early education performance of bereaved children^5,6^ may deserve further attention because it is associated with subsequent adverse socioeconomic trajectories and health outcomes.^4,7,8,9^

Losing a parent in childhood is highly stressful. Some bereaved children may develop complicated grief and posttraumatic stress,^10^ low stress resilience,^11^ long-lasting depressive symptoms,^12^ a sense of meaninglessness,^10^ and high-risk behaviors,^13^ leading to lower educational aspiration and achievement, even where university education is free.^5,6,14,15,16,17^ Bereaved children also lose human and social capital provided by the deceased parents, an important resource for child development.^18^ Furthermore, grief and mourning of the remaining parent^19^ may further reduce parental capacity of support and supervision for the child.

Previous research indicates that familial factors (eg, parental provision of cognitive stimulation) may partly account for the association.^5^ Sibling comparisons have been used to investigate how educational outcomes differ by age at parental death, assuming a linear trend.^20,21,22^ However, to the best of our knowledge, no study has compared compulsory school outcomes between siblings who were exposed to parental death before and after finishing compulsory school.

How educational consequences may differ by the age of the child at the time of parental death remains unclear. Younger age at parental death leads to a greater cumulative deficit of parental support and over a larger part of childhood, possibly associated with worse outcomes.^20^ Alternatively, parental death at certain developmental stages may be more impactful, eg, in periods of rapid growth and transitions.^23,24,25^ Therefore, relaxing the linear trend assumption will help to better understand the differential consequences of age at loss. Furthermore, previous studies suggested that bereaved children have higher risk of school failure than nonbereaved children, which may require more investigations.^5,15^

With the use of a national birth cohort and sibling comparison designs, this study aimed to investigate the association between childhood parental death and compulsory school outcomes at age 15 to 16 years, accounting for familial confounding, and whether children at certain ages were particularly vulnerable to the loss.

## Methods

### Study Population

**Figure 1.**
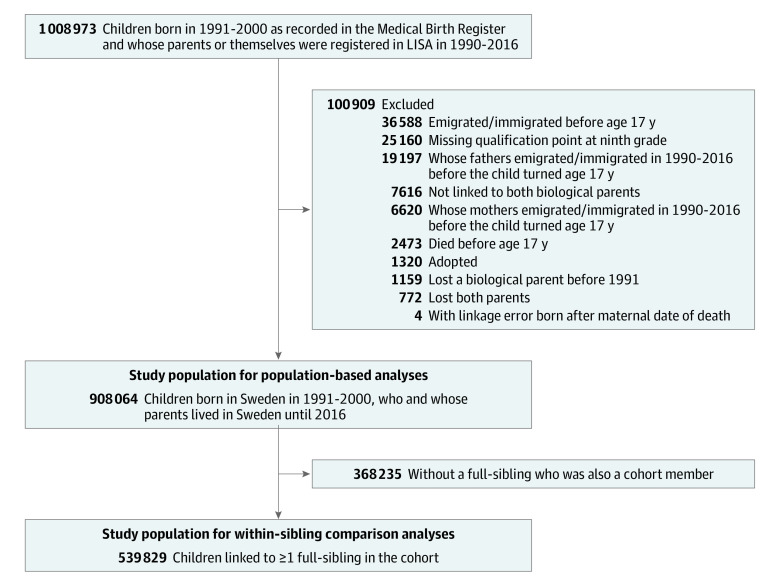
Flowchart of Study Population of 1991-2000 Birth Cohort LISA indicates Longitudinal Integrated Database for Health Insurance and Labor Market Studies.

All children born in Sweden between 1991 and 2000 were identified through the Swedish Medical Birth Register (N = 1 008 973) and linked to data on their parents, through the unique personal identifier^26^ and the Multi-Generation Register.^27^ We excluded children who were adopted, with missing data on both biological parents, who died or had an emigration or immigration record before reaching age 17 years, or whose parent had an emigration or immigration record before the child reached age 17 years. We also excluded children who lost both parents during the observation period 1990 to 2016 (n = 772, Figure 1). They may be adopted or placed in foster care, thus experiencing vastly different living circumstances than those with a remaining parent. A final birth cohort of 908 064 was used for population-based analyses using conventional regression models. The Ethical Review Board of Lund approved this study and waived the need for informed consent. Informed consent was waived because the data were anonymized. This cohort study followed the Strengthening the Reporting of Observational Studies in Epidemiology (STROBE) reporting guidelines. Data on race and ethnicity were not available as they were not collected in the Swedish registers.

With the unique personal identifier, information on each child in the final birth cohort and their parents was linked to the following national registers: Longitudinal Integrated Database for Health Insurance and Labor Market Studies (LISA), the National Patient Register, the Total Population Register, and the National School Register. All data were anonymized before we received them and cannot be used to identify any individual. All processing of data is regulated in the General Data Protection Regulation (EU) 2016/679.

We used a nested sibling subsample composed of all full siblings in the final birth cohort for within-sibling comparisons (539 829 children from 250 251 families) (Figure 1).

### Exposures

In conventional population-based analyses of the birth cohort sample, all cohort members who lost a parent before reaching age 17 years (ie, before or around graduating compulsory school at age 15 to 16 years) were categorized as the bereaved (n = 22 634). They were compared with nonbereaved children, including those never bereaved during the observation period or bereaved at 17 years or older (n = 885 430). The bereaved group was further classified into the paternally bereaved (n = 15 922) and maternally bereaved (n = 6712).

In the within-sibling comparison analysis, 10 934 children who lost a parent before turning age 17 years were compared with their nonexposed siblings, ie, their older siblings who also experienced parental death but only after having finished compulsory school (n = 7194). All 521 701 nonbereaved children who had a sibling in the final birth cohort were retained in the analysis as the nonexposed group. These nonbereaved siblings did not contribute to the estimation for exposure to parental death, but they informed the adjustment for confounding, such as the period and birth order.^22^

### Outcomes

Mean grades and ineligibility for upper secondary education (ineligible/eligible) were collected from the National School Register from 2006 to 2016 when the cohort members graduated from the compulsory school at age 15 to 16 years. Mean grade point summarizes the grades of the student’s 16 best-performing subjects from the final compulsory school year and ranges from 0 to 320. To be eligible for upper secondary education, students need passing grades in the core subjects (Swedish, English, and Mathematics) in the final year. It was then standardized into a *z* score (range, −3 to 3) among all students who graduated in the same year in Sweden.

### Confounders

We collected information on sex and date of birth (imputed date as the first day of the birth month) from the Medical Birth Register. Birth order is a nonshared confounder^28^ and was measured based on all mother-child linkages from the Multigeneration Register (1, 2, 3, 4, 5, and ≥6). Children who died before 1990 were not counted in birth order because they were not included in the linked database acquired for this study. We collected maternal and paternal education (basic, upper secondary, and tertiary) and household income of the mother in the year before the childbirth (quintile among all births in the same year) from the LISA database.

Linkages to the National Patient Register provided information on the maternal and paternal number of days of hospitalization for nonobstetric reasons in the year before birth (0 days, ≤1 week, >1 week to 1 month, or >1 month) as an indicator of parental health conditions preceding the exposure. From the Total Population Register, we collected country of birth status of the parents (both Swedish-born, mother born outside Sweden, father born outside Sweden, and both parents born outside Sweden) and calculated maternal and paternal age in the year of birth (continuous).

### Potential Modifiers

The consequences of parental death on the child may differ by the parental sociodemographic characteristics and the cause of parental death. Therefore, we performed exposure breakdown analyses by restricting on sex of the deceased parent or cause of the parental death, which was categorized into natural cause, accident, and suicide (eTable 1 in the Supplement provides the specific *International Classification of Diseases, Ninth Revision* and *International Statistical Classification of Diseases, Tenth Revision* codes). Furthermore, we stratified analyses by sex of the child, mother’s educational level, mother’s birth country, and mother’s specialized psychiatric health care use from either outpatient or inpatient care (assessed across the entire observation period 1990-2016 and dichotomized into no vs ever use).

### Statistical Analysis

Data analyses were performed on July 14, 2021. For the conventional population-based analyses, ordinary least-squares linear regression models were used to compare bereaved and nonbereaved children and to obtain their mean difference in the *z* score standardized mean school grades with 95% CIs. Modified Poisson regression models were used to estimate the risk ratios (RRs) with 95% CIs of ineligibility for upper secondary education associated with parental death, which was considered more preferable than an odds ratio when the outcome is not rare.

In within-sibling comparison analyses using the nested subsample, we used fixed-effect linear or fixed-effect Poisson regression models to evaluate the associations of parental death before turning age 17 years (yes or no) with *z* score school grades or ineligibility. Fixed-effect models automatically adjusted for both measured and unmeasured confounders that are shared and invariant between siblings. Therefore, we included only confounders that varied among siblings: sex, year of birth, birth order, household income of the mother in the year before the birth of the child, and maternal and paternal days of hospitalization in the year before birth.

To evaluate age at parental death in association with the school outcomes, age was modeled as a categorical variable, and each age (from 8 to 16 years) was compared with 17 years or older. Siblings born more than 10 years apart are not included because all birth cohort members were born between 1991 and 2000. The Wald test was used to examine whether there was a linear trend by age in the association. Statistical significance was set at *P* < .05, and all *P* values were 2-sided. All analyses were performed using Stata, version MP 15.1 (StataCorp LLC).

To extend the age of bereavement to a wider range (from 0 to 16 years, compared with ≥17 years), we formed a complementary sibling sample by adding all of their older siblings born in 1983 to 1990 to the final birth cohort (n = 889 729) and selecting all the full siblings from this expanded birth cohort. These additional siblings could not be included in the main analyses because their data before 1990 were not available and we were unable to identify a few confounders in the year before their births. Thus, in the complementary analysis with these siblings, we used the information closest (in 1990) to their birth. Siblings born before 1983 were not added into the complementary sample because they finished compulsory education when the grading system was different. In all models, cluster robust estimation for standard error was used to account for associations among siblings.

## Results

For the 908 064 children in the full birth cohort, the mean (SD) age in 2016 (end of observation) was 20.9 (2.9) years; for the 889 729 children in the complementary sibling sample, mean (SD) age in 2016 was 23.1 (3.9) years. The bereaved children, that is, those who lost a parent before turning age 17 years (N = 22 634; 2.5% of the final birth cohort; 11 553 boys [51.0%]; 11 081 girls [49.0%]; mean [SD] age, 21.0 [2.8] years), were more likely to be born in the earlier years of the cohort, were more likely to have parents who were born outside Sweden, with lower educational level, lower income, and higher morbidity before the child was born compared with children who had not lost a parent (Table 1). The sex distribution was similar among the bereaved and nonbereaved children.

**Table 1.** Characteristics of 1991-2000 Birth Cohort (N = 908 064)

Characteristic	No. (column %)		
	All (N = 908 064)	No parental death before finishing school (n = 885 430)	Parental death before finishing school (n = 22 634)
Mean qualification point, mean (SD)	213.8 (62.7)	214.3 (62.4)	193.8 (71.0)
Year of birth			
1991-1995	515 784 (56.8)	502 586 (56.8)	13 198 (58.3)
1996-2000	392 280 (43.2)	382 844 (43.2)	9436 (41.7)
Sex			
Girl	443 608 (48.9)	432 527 (48.8)	11 081 (49.0)
Boy	464 456 (51.1)	452 903 (51.2)	11 553 (51.0)
Birth order			
First born	368 231 (40.6)	360 410 (40.7)	7806 (34.5)
Second or third born	479 665 (52.8)	467 747 (52.8)	11 924 (52.7)
Fourth or higher	60 168 (6.6)	57 273 (6.5)	2904 (12.8)
Maternal educational level (in the year before birth)			
Basic	162 813 (17.9)	156 707 (17.7)	6106 (27.0)
Upper secondary	341 542 (37.6)	333 041 (37.6)	8501 (37.6)
Tertiary	403 709 (44.5)	395 682 (44.7)	8027 (35.5)
Paternal educational level (in the year before birth)			
Basic	184 762 (20.3)	177 950 (20.1)	6812 (30.1)
Upper secondary	370 021 (40.7)	361 219 (40.8)	8802 (38.9)
Tertiary	353 281 (38.9)	346 261 (39.1)	7020 (31.0)
Parental country of birth			
Both Swedish born	735 174 (81.0)	718 184 (81.1)	16 990 (75.1)
Born outside Sweden			
Mother	42 449 (4.7)	40 937 (4.6)	1512 (6.7)
Father	47 424 (5.2)	45 794 (5.2)	1630 (7.2)
Both	83 017 (9.1)	80 515 (9.1)	2502 (11.1)
Disposable family income per person (mother in the year before birth)			
Highest quintile	181 410 (20)	177 816 (20.1)	3594 (15.9)
Second	180 676 (19.9)	176 977 (20.0)	3699 (16.3)
Third	179 827 (19.8)	175 785 (19.9)	4042 (17.9)
Fourth	177 812 (19.6)	172 938 (19.5)	4874 (21.5)
Lowest quintile	188 339 (20.7)	181 914 (20.5)	6425 (28.4)
Maternal total No. of days in hospital in the year before birth			
0	794 181 (87.5)	775 140 (87.5)	19 041 (84.1)
≤1 wk	88 314 (9.7)	85 747 (9.7)	2567 (11.3)
>1 wk and ≤1 mo	21 874 (2.4)	21 031 (2.4)	843 (3.7)
>1 mo	3695 (0.4)	3512 (0.4)	183 (0.8)
Paternal total No. of days in hospital in the year before birth			
0	871 059 (95.9)	850 421 (96.0)	20 638 (91.2)
≤1 wk	31 019 (3.4)	29 694 (3.4)	1325 (5.9)
>1 wk and ≤1 mo	5204 (0.6)	4691 (0.5)	513 (2.3)
>1 mo	782 (0.1)	624 (0.1)	158 (0.7)

Table 2 shows the results from the conventional population-based analyses of the association between bereavement and school outcomes. Compared with the nonbereaved children, bereaved children had lower mean grade *z* scores (adjusted β coefficient, −0.19; 95% CI, −0.21 to −0.18; *P* < .001) and higher risk of ineligibility for upper secondary education (adjusted RR, 1.36; 95% CI, 1.32-1.41; *P* < .001). The association was slightly stronger in paternally bereaved children than maternally bereaved children.

**Table 2.** Population-Based Analyses on the Association Between Parental Death and Eligibility for Attending Secondary Education and Mean School Grades Among 908 064 Swedish Children^a^

Variable	No.	School outcomes in the final year of compulsory school at age 15-16 y				
		Ineligibility for upper secondary education			Mean grade *z* score, β coefficient (95% CI)	
		No. (%)	Risk ratio (95% CI)			
			Unadjusted	Adjusted risk ratio	Unadjusted	Adjusted
Parental death before finishing school						
Parental death	22 634	3570 (15.8)	1.74 (1.68 to 1.80)^b^	1.36 (1.32 to 1.41)^b^	−0.30 (−0.32 to −0.29)^b^	−0.19 (−0.21 to −0.18)^b^
No parental death	885 430	80 241 (9.1)	1 [Reference]	1 [Reference]	0 [Reference]	0 [Reference]
Maternal death before finishing school						
Maternal death	6712	927 (13.8)	1.52 (1.43 to 1.63)^b^	1.31 (1.23 to 1.39)^b^	−0.23 (−0.26 to −0.20)^b^	−0.17 (−0.19 to −0.15)^b^
No parental death	885 430	80 241 (9.1)	1 [Reference]	1 [Reference]	0 [Reference]	0 [Reference]
Paternal bereavement before finishing school						
Paternal death	15 922	2643 (16.6)	1.83 (1.76 to 1.90)^b^	1.38 (1.33 to 1.43)^b^	−0.33 (−0.35 to −0.31)^b^	−0.20 (−0.22 to −0.19)^b^
No parental death	885 430	80 241 (9.1)	1 [Reference]	1 [Reference]	0 [Reference]	0 [Reference]

^a^ Poisson regression was used for eligibility, and ordinary least square linear regression was used for mean grade *z* score. Cluster robust estimation for standard error was used to account for associations between siblings. In both regression models, adjusted estimates were obtained from models described in the Methods section.

^b^ Statistical significance at the *P* < .05 level.

Table 3 shows the results from analyses of the same association but using the within-sibling comparison design. Children who experienced parental death before age 17 years showed a lower mean grade *z* score compared with their older siblings who experienced the death after finishing compulsory school (adjusted β coefficient, −0.06; 95% CI, −0.10 to −0.01; *P* = .02). However, children exposed before finishing school did not have a significantly higher risk of ineligibility for upper secondary education than their siblings (adjusted RR, 1.07; 95% CI, 0.93-1.24; *P* = .34). The results were consistent in the complementary sibling sample that included the older siblings of the cohort members (adjusted β coefficient, −0.05; 95% CI, −0.08 to −0.02; *P* = .001 and adjusted RR, 1.08; 0.99-1.18; *P* = .08). To assess the generalizability of the within-sibling comparison sample, we compared children with and without a full sibling, and the characteristics and associations appeared similar (eTables 2, 3, and 4 in the Supplement).

**Table 3.** Within-Sibling Comparison of School Outcomes of 539 829 Children Exposed to Parental Death Before Graduating From Compulsory School Compared With Their Siblings Exposed to the Death After Graduation^a^

Variable	No.	School outcomes in the final year of compulsory school at age 15-16 y		
		Ineligibility for upper secondary education		Mean grade *z* score, adjusted β coefficient (95% CI)
		No. (%)	Adjusted risk ratio	
Parental death before finishing school	10 934	1587 (14.5)	1.07 (0.93 to 1.24)	−0.06 (−0.10 to −0.01)^b^
No parental death	528 895	43 161 (8.2)	1 [Reference]	0 [Reference]

^a^ Fixed-effect Poisson regression was used for eligibility, and fixed-effect linear regression was used for mean grade *z* score. Cluster robust estimation for standard error was used to account for associations between siblings. Adjusted estimates were obtained from models described in the Methods section.

^b^ Statistical significance at the *P* < .05 level.

**Figure 2.**
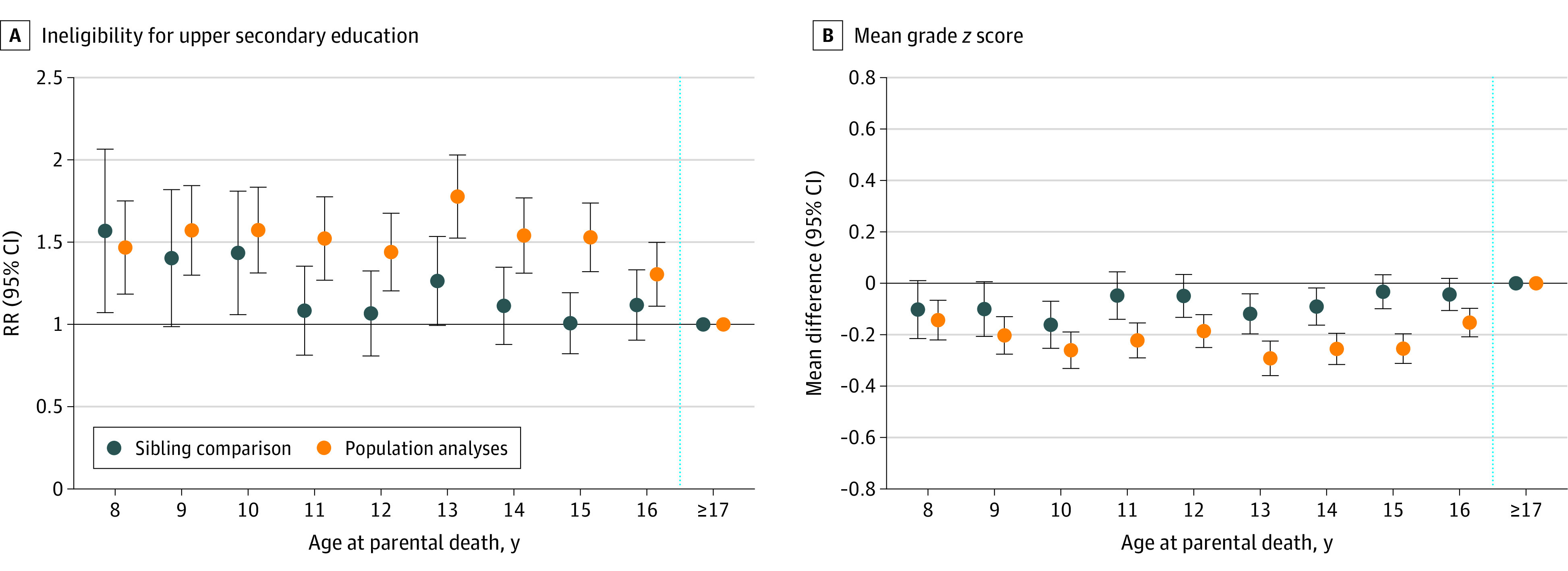
Population-Based Analyses and Within-Sibling Comparison of School Outcomes by Age at Parental Death Among 539 829 Swedish Children The point estimations from the within-sibling comparison are shown as dark blue data markers with 95% confidence intervals. Fixed-effect Poisson regression was used for eligibility (A), and fixed-effect linear regression was used for mean grade *z* score (B). For comparison, the point estimations from population-based analyses using conventional ordinary least square linear and Poisson regression models are also presented as orange data markers. All models adjusted for sex, year of birth, birth order, maternal household income in the year before birth, and maternal and paternal days of hospitalization in the year before birth. Cluster robust estimation for standard error was used to account for associations between siblings. The blue vertical dashed line indicates the age by which the school outcome had been measured. The horizontal line indicates the reference of children who lost a parent at age 17 years or older.

To explore the age pattern of the associations, we plotted the mean differences in mean grade *z* score and ineligibility for upper secondary education by age at parental death (ages 8-16 years), in reference to their siblings who lost a parent at 17 years or older (Figure 2). In the conventional population-based analyses on the nested sibling subsample, the association between parental death and school outcomes tended to be stronger for individuals who experienced parental death in the older ages (age 13 to 16 years). In contrast, within-sibling analysis on the same sample showed a linear age trend, where a younger age at parental death was associated with a lower mean grade *z* score. Ineligibility showed no linear age trend, corresponding to the overall null association within siblings.

With the use of the complementary sibling sample, conventional population-based analyses also showed stronger associations at age 13 to 15 years. However, within-sibling analyses on the complementary sibling sample did not confirm the linear age trend for mean grade *z* score (eFigure in the Supplement).

The association between parental death and school grades appeared more pronounced among children who experienced paternal deaths, deaths owing to natural causes, or of a mother with tertiary education (eTable 5 in the Supplement). Of note, experiencing maternal death before age 17 years was not associated with reduced mean school grades within siblings (adjusted β coefficient, 0.00; 95% CI, −0.08 to 0.08). Boys and girls showed similar associations. The mother having been born outside of Sweden or having had specialized psychiatric health care did not seem to modify the association. Consistent with the main analysis, parental death was not associated with ineligibility for upper secondary education in any of the strata.

## Discussion

Using a within-sibling comparison design controlling for shared familial confounding, we found that parental death, especially paternal death, was associated with lower mean school grades at finishing compulsory school. Children who lost a parent at a younger age tended to have lower mean school grades than their older siblings.

Although the association between parental bereavement and education outcomes has been described previously,^5,15^ a sibling comparison design has only been used to evaluate the differential association of age at parental death and educational outcomes.^20,21,22^ None of these studies took advantage of the sibling design to evaluate whether education outcomes were associated with parental death. Therefore, to measure the association of parental death itself with school outcomes, we used a unique approach to classify siblings into 2 groups, exposed to parental death before or after the age of completing compulsory school, taking advantage of the sibling comparison design to adjust for measured and unmeasured familial confounding. Furthermore, by including the nonbereaved siblings in the model, the association was also independent of the birth order or period that may explain differences between siblings.

Sibling comparisons showed that childhood bereavement was associated with lower mean school grades but with much weaker associations than in the population analyses. These findings may indicate 2 mechanisms. First, the reduced associations in the within-sibling comparisons suggested that unmeasured familial factors that were shared between siblings largely explained the difference in school outcomes between bereaved and nonbereaved children. Psychosocial environments, such as parenting attitudes and support, may be crucial for school outcomes,^29^ although such familial factors could not be identified in our analyses. Second, the smaller but remaining association in the sibling comparison suggested that parental death was associated with lower school grades, even after adjustment for shared unmeasured confounding or a wide range of measured confounding that varied between siblings.

Unlike school grades, the risk of ineligibility for upper secondary education did not increase after parental death in the sibling analyses. It is likely that the difference in school grades was not strong enough to affect passing or failing in the 3 core subjects that determined eligibility. Another explanation could be the differently selected sibling samples because only siblings with discordant outcomes contributed to the estimation for ineligibility.

Children who lost a parent at a younger age may lose a source of important parental social capital^18^ and subsequent supervision, support, and control over school work to a greater extent than their older siblings. Our analysis on the nested sibling subsample suggested a greater lifetime loss of parental support associated with losing a parent at a younger age. A similar age pattern was found in the association between parental loss and university attainment.^20^ However, given the inconclusive age pattern from the 2 sibling samples in our analyses, future studies may use a larger sequential birth cohort observed over a more extended period to verify our findings.

The association between paternal death and school grades was stronger than maternal death. This may suggest 2 possibilities: that fathers play essential roles in child development and educational achievement,^30^ or that experiences of widows and widowers differ, which in turn differentially affect the bereaved child. On the one hand, paternal death may entail a greater socioeconomic consequence owing to the gender pay gap, although such association may be largely alleviated by financial support available for the bereaved family in Nordic countries.^21^ On the other hand, even though the association between spousal death and depression is suggested to be stronger among men than women, bereaved women may be more expressive of their grief.^31^ Paternal death may have a greater indirect association through the mother’s grieving. Therefore, supporting the bereaved family, especially the remaining parent, may be crucial for improving the child's developmental outcomes.

The study used birth cohort data based on several national registers with high levels of completeness and minimal loss to follow-up.^32,33,34^ The national registers also provided opportunities to link family members and prospectively measured parents’ health and social variables before childbirth. Using fixed-effects models for within-sibling comparison, we could adjust for measured and unmeasured confounding that were shared among siblings.

### Limitations

The limitations of this study include confounding by factors that were not shared between siblings.^35^ To minimize such confounding, we adjusted a rich set of confounders measured from before the exposure occurred. Without the date of finishing school in each child, the association at age 15 to 16 years could have been underestimated when some of the children in this category may have in fact experienced parental death after finishing school, thus being erroneously classified as exposed when in fact being unexposed. Also, sibling analysis on the association at a very young age could only be evaluated from selected sets of siblings with unusually large age gaps among them. With a social welfare system providing generous supports for children, the impacts of parental death may be less strong in Sweden than in other settings.

## Conclusions

The findings of this cohort study indicate that parental death before finishing compulsory education was associated with lower school grades, independent of familial confounding shared between siblings. Children who have lost parents may benefit from additional educational support in school. Thus, further research is needed to better understand the factors that mediate the association to appropriately support the children at risk for school challenges. This might reduce the risk of adverse adult socioeconomic trajectories in children who lost a parent before completing compulsory schooling.

## Notes

Supplement. **eTable 1.** ICD-9 and ICD-10 Codes for Categories of Causes of Death **eTable 2.** Characteristics of the Children With and Without Full-Siblings in the Cohort (N = 908,064) **eTable 3.** Population-Based Analyses on the Association Between Parental Death and School Outcomes Among Children Having a Full-Sibling in the Cohort (N = 539,829) **eTable 4.** Population-Based Analyses on the Association Between Parental Death and School Outcomes Among Children Not Having a Full-Sibling in the Cohort (N = 368,235) **eTable 5.** Within-Sibling Comparison of School Outcomes of Children Exposed to Parental Death Before Graduating From Compulsory School Compared With Their Siblings Exposed to the Death After Graduation, Stratified by the Potential Modifiers **eFigure.** Within-Sibling Comparison of School Outcomes by Age at Parental Death on the Complementary Sibling Sample (N = 889,729)
