# Supplementary material for: School Outcomes Among Children Following Death of a Parent
Source: JAMA Netw Open. 2022 Apr 8;5(4):e223842. doi: 10.1001/jamanetworkopen.2022.3842 (PMC8994122; doi:10.1001/jamanetworkopen.2022.3842)
Supplement: Supplement. — eTable 1. ICD-9 and ICD-10 Codes for Categories of Causes of Death eTable 2. Characteristics of the Children With and Without Full-Siblings in the Cohort (N = 908,064) eTable 3. Population-Based Analyses on the Association Between Parental Death and School Outcomes Among Children Having a Full-Sibling in the Cohort (N = 539,829) eTable 4. Population-Based Analyses on the Association Between Parental Death and School Outcomes Among Children Not Having a Full-Sibling in the Cohort (N = 368,235) eTable 5. Within-Sibling Comparison of School Outcomes of Children Exposed to Parental Death Before Graduating From Compulsory School Compared With Their Siblings Exposed to the Death After Graduation, Stratified by the Potential Modifiers eFigure. Within-Sibling Comparison of School Outcomes by Age at Parental Death on the Complementary Sibling Sample (N = 889,729) [file jamanetwopen-e223842-s001.pdf]

Supplemental Online Content

Liu C, Grotta A, Hiyoshi A, Berg L, Rostila M. School outcomes among children following death of a parent. *JAMA Netw Open*. 2022;5(4):e223842. doi:10.1001/jamanetworkopen.2022.3842

eTable 1. ICD-9 and ICD-10 Codes for Categories of Causes of Death

eTable 2. Characteristics of the Children With and Without Full-Siblings in the Cohort (N=908,064)

eTable 3. Population-Based Analyses on the Association Between Parental Death and School Outcomes Among Children Having a Full-Sibling in the Cohort (N=539,829)

eTable 4. Population-Based Analyses on the Association Between Parental Death and School Outcomes Among Children Not Having a Full-Sibling in the Cohort (N=368,235)

eTable 5. Within-Sibling Comparison of School Outcomes of Children Exposed to Parental Death Before Graduating From Compulsory School Compared With Their Siblings Exposed to the Death After Graduation, Stratified by the Potential Modifiers

eFigure. Within-Sibling Comparison of School Outcomes by Age at Parental Death on the Complementary Sibling Sample (N = 889,729)

This supplemental material has been provided by the authors to give readers additional information about their work.

© 2022 Liu C et al. *JAMA Network Open.*

eTable 1. ICD-9 and ICD-10 codes for categories of causes of death

Natural cause of death	ICD-9 code: 000-796; and ICD-10 code: A00-R99	If the cause was not determined as accident or suicide, the disease cause of death was determined based on the "Main cause of death".
Accident	ICD-9 code: 800-999; and ICD-10 code: V01-Y98	If the death was not suicide, the external cause was determined based on the "Main cause of death".
Suicide	ICD 9: E950–E959, E980–E989; ICD-10: X60–X84, Y10–Y34	Suicide was determined if any of the recorded "Main cause of death" or the "Contributing cause of death" was suicide.

© 2022 Liu C et al. *JAMA Network Open.*

eTable 2. Characteristics of the children with and without full-siblings in the cohort (N=908,064)

	**All**	**Having a full-sibling in the cohort**	**Excluded due to having no full-sibling in the cohort**
	**N = 908,064**	**N=539,829**	**N=368,235**
	**n (Col %)**	**n (Col %)**	**n (Col %)**
Mean qualification point, Mean (SD)	213.8 (62.7)	216.9 (61.0)	209.2 (64.8)
Year of birth			
1991-1995	515784 (56.8)	303663 (56.3)	212121 (57.6)
1996-2000	392280 (43.2)	236166 (43.7)	156114 (42.4)
Gender			
Girl	443608 (48.9)	263170 (48.8)	180438 (49.0)
Boy	464456 (51.1)	276659 (51.2)	187797 (51.0)
Birth order			
1st born	368231 (40.6)	196820 (36.5)	171411 (46.5)
2nd-3rd born	479665 (52.8)	308303 (57.1)	171362 (46.5)
4th or higher	60168 (6.6)	34706 (6.4)	25462 (6.9)
Maternal education (in the year before birth)			
Basic	162813 (17.9)	83883 (15.5)	78930 (21.4)
Upper secondary	341542 (37.6)	203332 (37.7)	138210 (37.5)
Tertiary	403709 (44.5)	252614 (46.8)	151095 (41.0)
Paternal education (in the year before birth)			
Basic	184762 (20.3)	98892 (18.3)	85870 (23.3)
Upper secondary	370021 (40.7)	223383 (41.4)	146638 (39.8)
Tertiary	353281 (38.9)	217554 (40.3)	135727 (36.9)
Parental foreign-born status			
Both Swedish born	735174 (81.0)	442975 (82.1)	292199 (79.4)
Mother foreign born	42449 (4.7)	22084 (4.1)	20365 (5.5)
Father foreign born	47424 (5.2)	26267 (4.9)	21157 (5.7)
Both foreign born	83017 (9.1)	48503 (9.0)	34514 (9.4)
Disposable family income per person (mother in the year before birth)			
Highest quintile	181410 (20.0)	109849 (20.3)	71561 (19.4)
Second	180676 (19.9)	110665 (20.5)	70011 (19.0)
Third	179827 (19.8)	110383 (20.4)	69444 (18.9)
Fourth	177812 (19.6)	103994 (19.3)	73818 (20.0)
Lowest quintile	188339 (20.7)	104938 (19.4)	83401 (22.6)
Maternal total number of days in hospital in the year before birth			
0	794181 (87.5)	473878 (87.8)	320303 (87)
<=1 week	88314 (9.7)	51460 (9.5)	36854 (10.0)
>1 week and <=1 month	21874 (2.4)	12410 (2.3)	9464 (2.6)
> 1 month	3695 (0.4)	2081 (0.4)	1614 (0.4)

© 2022 Liu C et al. *JAMA Network Open.*

	All	Having a full-sibling in the cohort	Excluded due to having no full-sibling in the cohort
	**N = 908,064**	**N=539,829**	**N=368,235**
	**n (Col %)**	**n (Col %)**	**n (Col %)**
Paternal total number of days in hospital in the year before birth			
0	871059 (95.9)	518959 (96.1)	352100 (95.6)
<=1 week	31019 (3.4)	17776 (3.3)	13243 (3.6)
>1 week and <=1 month	5204 (0.6)	2767 (0.5)	2437 (0.7)
> 1 month	782 (0.1)	327 (0.1)	455 (0.1)

© 2022 Liu C et al. *JAMA Network Open.*

eTable 3. Population-based analyses on the association between parental death and school outcomes among children having a full-sibling in the cohort (N=539,829)

	**N obs**	**School outcomes in the final year of compulsory school at age 15-16**				
		**Ineligibility for upper-secondary education**			**Mean grade z-score**	
		**n (%)**	**Unadjusted risk ratio**	**Adjusted risk ratio**	**Unadjusted beta coefficient**	**Adjusted beta coefficient**
Parental bereavement before graduation						
Bereaved	10,934	1587 (14.5)	**1.78 [1.69,1.88]**	**1.36 [1.29,1.43]**	**-0.29 [-0.32,-0.27]**	**-0.19 [-0.21,-0.17]**
Non-bereaved	528,895	43,162 (8.2)	1.00 [Reference]	1.00 [Reference]	0.00 [Reference]	0.00 [Reference]
Maternal bereavement before graduation						
Bereaved	3,300	428 (13.0)	**1.59 [1.43,1.76]**	**1.34 [1.21,1.48]**	**-0.21 [-0.25,-0.17]**	**-0.15 [-0.19,-0.12]**
Non-bereaved	528,895	43,162 (8.2)	1.00 [Reference]	1.00 [Reference]	0.00 [Reference]	0.00 [Reference]
Paternal death before graduation						
Bereaved	7,634	1159 (15.2)	**1.86 [1.75,1.98]**	**1.37 [1.29,1.45]**	**-0.33 [-0.36,-0.30]**	**-0.20 [-0.23,-0.18]**
Non-bereaved	528,895	43,162 (8.2)	1.00 [Reference]	1.00 [Reference]	0.00 [Reference]	0.00 [Reference]

Poisson regression was used for eligibility, and OLS linear regression was used for mean grade z-score. Cluster robust estimation for standard error was used to account for correlations between siblings. In both regression models, adjusted estimates were obtained from models including gender, year of birth, parental foreign-born status, maternal age in the year of birth, maternal and paternal education in the year before birth, maternal household income in the year before birth, maternal and paternal days of hospitalisation in the year before birth. Bold values denote statistical significance at the $P < 0.05$ level.

© 2022 Liu C et al. *JAMA Network Open.*

eTable 4. Population-based analyses on the association between parental death and school outcomes among children not having a full-sibling in the cohort (N=368,235)

	N obs	School outcomes in the final year of compulsory school at age 15-16				
		Ineligibility for upper-secondary education			Mean grade z-score	
		n (%)	Unadjusted risk ratio	Adjusted risk ratio	Unadjusted beta coefficient	Adjusted beta coefficient
Parental bereavement before graduation						
Bereaved	11,700	1983 (16.9)	**1.63 [1.56,1.70]**	**1.34 [1.28,1.39]**	**-0.29 [-0.30,-0.27]**	**-0.19 [-0.20,-0.17]**
Non-bereaved	356,535	37,082 (10.4)	1.00 [Reference]	1.00 [Reference]	0.00 [Reference]	0.00 [Reference]
Maternal bereavement before graduation						
Bereaved	3,412	499 (14.6)	**1.41 [1.30,1.53]**	**1.25 [1.16,1.36]**	**-0.22 [-0.25,-0.19]**	**-0.17 [-0.20,-0.14]**
Non-bereaved	356,535	37,082 (10.4)	1.00 [Reference]	1.00 [Reference]	0.00 [Reference]	0.00 [Reference]
Paternal death before graduation						
Bereaved	8,288	1484 (17.9)	**1.72 [1.64,1.80]**	**1.36 [1.30,1.43]**	**-0.31 [-0.34,-0.29]**	**-0.19 [-0.21,-0.17]**
Non-bereaved	356,535	37,082 (10.4)	1.00 [Reference]	1.00 [Reference]	0.00 [Reference]	0.00 [Reference]

Poisson regression was used for eligibility, and OLS linear regression was used for mean grade z-score. Cluster robust estimation for standard error was used to account for correlations between siblings. In both regression models, adjusted estimates were obtained from models including gender, year of birth, parental foreign-born status, maternal age in the year of birth, maternal and paternal education in the year before birth, maternal household income in the year before birth, maternal and paternal days of hospitalisation in the year before birth. Bold values denote statistical significance at the $P < 0.05$ level.

© 2022 Liu C et al. *JAMA Network Open.*

eTable 5. Within-sibling comparison of school outcomes of children exposed to parental death before graduating from compulsory school compared with their siblings exposed to the death after graduation, stratified by the potential modifiers

	**N observation**	**Ineligibility for upper-secondary education**	**Mean grade z-score**
		**Adjusted risk ratio**	**Adjusted beta coefficient**
Restricted on the gender of the deceased parent*			
Maternal death	527337	0.97 [0.72,1.31]	0.00 [-0.08,0.08]
Paternal death	534193	1.11 [0.95,1.03]	**-0.08 [-0.14,-0.03]**
Restricted on the cause of parental death*			
Natural cause	535258	1.12 [0.95,1.31]	**-0.06 [-0.11,-0.01]**
Accident	523695	0.75 [0.42,1.35]	-0.05 [-0.20,0.10]
Suicide	524274	1.01 [0.70,1.43]	-0.04 [-0.17,0.09]
Stratified by gender of the child			
Girl	263170	1.28 [0.95,1.73]	-0.06 [-0.14,0.03]
Boy	276659	1.09 [0.87,1.37]	-0.07 [-0.16,0.01]
Stratified by maternal education			
Basic	83883	1.02 [0.83,1.26]	-0.05 [-0.17,0.07]
Upper-secondary	203332	1.11 [0.87,1.41]	-0.06 [-0.14,0.01]
Tertiary	252614	1.43 [0.95,2.16]	**-0.09 [-0.16,-0.02]**
Stratified by maternal birth country			
Swedish-born	469242	1.26 [0.97,1.63]	**-0.06 [-0.11,-0.01]**
Foreign-born	70587	1.02 [0.86,1.21]	-0.05 [-0.16,0.06]
Stratified by maternal psychiatric health			
No specialized psychiatric health care in 1991-2016	468114	1.04 [0.87,1.23]	**-0.06 [-0.11,-0.01]**
Having specialized psychiatric health care in 1991-2016	71715	1.18 [0.92,1.51]	-0.05 [-0.16,0.05]

Fixed effect Poisson regression was used for eligibility, and fixed effect linear regression was used for mean grade z-score. Cluster robust estimation for standard error was used to account for correlations between siblings. Adjusted estimates were obtained from models including gender, year of birth, birth order, maternal household income in the year before birth, maternal and paternal days of hospitalisation in the year before birth. Bold values denote statistical significance at the P < 0.05 level. *Restricting to all nonbereaved children and the bereaved children having deceased parents of the specified gender or causes of death.

© 2022 Liu C et al. *JAMA Network Open.*

eFigure. Within-sibling comparison of school outcomes by age at parental death on the complementary sibling sample (N = 889,729)

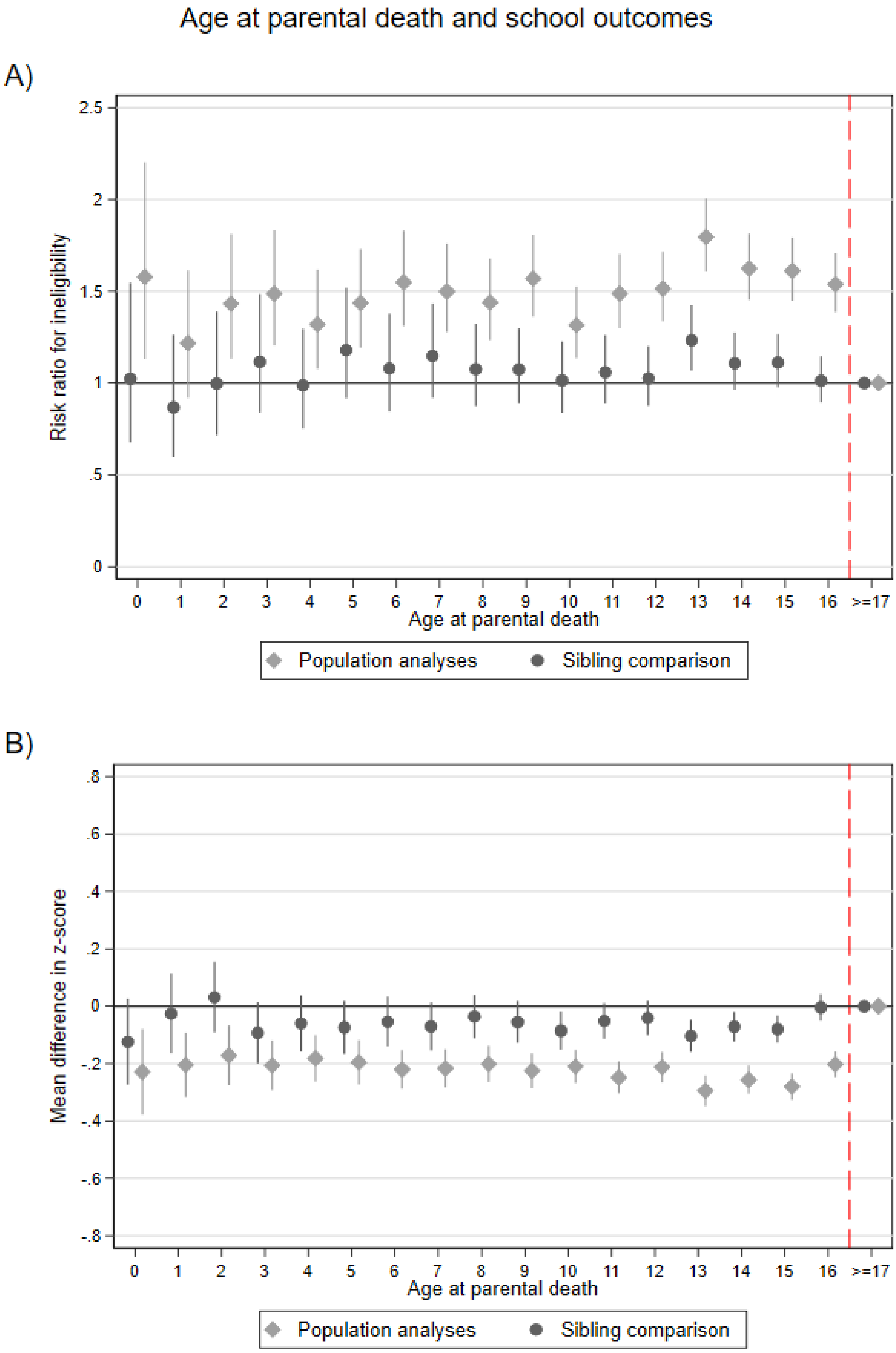

The point estimations from the within-sibling comparison are shown as black dots with 95% confidence intervals. Fixed effect Poisson regression was used for eligibility (panel A), and fixed effect linear regression was used for mean grade z-score (panel B). For comparison, the

© 2022 Liu C et al. *JAMA Network Open.*

point estimations from population-based analyses using conventional OLS linear and Poisson regression models are also presented as grey diamonds. All models adjusted for gender, year of birth, birth order, maternal household income in the year before birth, maternal and paternal days of hospitalisation in the year before birth. Cluster robust estimation for standard error was used to account for correlations between siblings. The red vertical dash line indicates the age by which the school outcome had been measured. The horizontal line indicates the reference of children who lost a parent at age 17 or older.

© 2022 Liu C et al. *JAMA Network Open.*
